# Effects of Salinity and Nutrient Addition on Mangrove *Excoecaria agallocha*

**DOI:** 10.1371/journal.pone.0093337

**Published:** 2014-04-01

**Authors:** Yaping Chen, Yong Ye

**Affiliations:** Key Laboratory of the Ministry of Education for Coastal and Wetland Ecosystem, College of the Environment and Ecology, Xiamen University, Xiamen, Fujian, China; United States Department of Agriculture, United States of America

## Abstract

Effects of salinity on seed germination and growth of young (1 month old) and old (2-year old) seedlings of *Excoecaria agallocha* were investigated. Combined effects of salinity and nutrient level were also examined on old seedlings. Seed germination was best at 0 and 5 psu salinity. 15 psu salinity significantly delayed root initiation and decreased final establishment rate. All seeds failed to establish at 25 psu salinity. Young seedlings performed best at 0 and 5 psu, but growth was stunned at 15 psu, and all seedlings died within 90 days at 25 psu. Old seedlings grew best at salinities below 5 psu and they survived the whole cultivation at 25 psu. This indicated that *E. agallocha* increased salt tolerance over time. Gas exchange was significantly compromised by salinities above 15 psu but evidently promoted by high nutrient. Proline accumulated considerably at high nutrient, and its contents increased from 0 to 15 psu but decreased at 25 psu salinity. Lipid peroxidation was aggravated by increasing salinity beyond 15 psu but markedly alleviated by nutrient addition. These responses indicated that *E. agallocha* was intolerant to high salinity but it can be greatly enhanced by nutrient addition.

## Introduction

Mangrove forests are distributed along coastlines and periodically inundated by seawater. The particularity of their habitat makes salinity an important factor limiting propagule germination, seedling growth and reproduction of mangrove trees [1]–[3]. Many studies dealt with the effects of salinity on mangroves. A negative relationship between seedling emergence rate and salt content was obtained in *Avicennia marina* [4]. Increasing salinity delays root initiation of *Acanthus ilicifolius* and reduces final seedling establishment rates of *Aegiceras corniculatum* [5], [6]. Moderate salinities stimulate growths of *Av. marina* [7] and *Ae. corniculatum* [8], but further increases in salinity inevitably decrease their growths. Biomass accumulation and stem elongation of *Sonneratia alba* and *S. lanceolata* show a tendency to be stimulated by low salinity and inhibited by further increasing salinity [9]. Medina and Francisco [10] obtained significant decreases in leaf number and area of *Av. germinans* as soil salinity increased. Under extreme salinity stress, accelerated leaf mortality rates of mangrove seedlings are often accompanied by decreases in leaf production rates, finally leading to the deaths of plants [9], [11], [12]. High salinity can cause osmotic stress and reduce the availability of water, resulting in stomatal closure and reduced supply of carbon dioxide [13], [14]. In addition, salt stress can induce ion toxicities such as membrane disorganization, production of reactive oxygen species, and disturbance of nutrient balance [4], [15], [16]. Increases in salinity reduce nitrogen accumulation in *Kandelia obovata* [17] and inhibit the uptake of K^+^ by *Av. marina*, resulting in damage to photosynthetic apparatus [4]. On the other hand, during long term of acclimation to saline conditions, mangroves evolve various strategies to cope with high salinity, including anatomical [18]–[20], physiological [21], and molecular [22], [23] mechanisms. Patel et al. [4] showed that with increases in salinity, *Av. marina* increased contents of leaf proline to alleviate NaCl stress. In order to defend salt-induced oxidative damage, plants are equipped with oxygen radical detoxifying enzymes such as superoxide dismutase, peroxidase and catalase [6], [24]. Accumulation of inorganic ions in vacuoles is common pattern observed in mangrove plants under saline conditions [25], [26], which serves not only to increase cellular osmolarity to counter osmotic stress but also to avoid increases in ionic strength of the cytoplasm [21], [27]. However, previous studies on this topic mostly focused on a certain growth stage of mangroves, how salinity influences mangroves in a dynamic developmental process is not well known.

Nutrient deficiency is another main problem limiting mangrove growth [28]–[30]. Different from most terrestrial soils, mangrove sediments are frequently waterlogged by seawater. Waterlogging results in anaerobic environment, which greatly restrains nitrification and consequently leads to low nutrient bioavailability of mangrove sediments [20], [31]. Nutrient addition significantly accelerates shoot elongation of *Rhizophora mangle* [32], increases leaf and branch growths of *Av. germinans* [33] and remarkably enlarges leaf area of *Ceriops tagal* [34]. Increases in nutrient availability evidently increase stomatal conductance and photosynthetic rate of *K. obovata* seedlings [17]. For *Av. germinans* trees, nutrient addition increases nitrogen investment in the osmotically compatible solute, glycine betaine, which improves water status of tissues and enhanced photosynthesis [35], [36]. Studies on plant anatomical mechanism demonstrated that nutrient addition might enhance water supply to leaves and increase hydraulic conductivity by stimulating root growth and/or improving some aspects of the water conducting pathway [1], [37], [38]. The applications of nutrient fertilizers were also reported to alleviate the phytotoxicity of heavy metals [20], [39], [40], contribute to osmoregulation [17], [41] and stimulate antioxidant systems [42], all of which endow plants with stronger resistance to adverse environments including prolonged waterlogging and extreme salinities.

Although the ecological and economic importance of mangrove forests are widely acknowledged, these special coastal ecosystems have for a long time been intensively affected by both anthropogenic activities and natural disasters, and suffered from significant habitat loss on a world wide scale [43]–[45]. Thus, restoring these fragmented ecosystems becomes an imperative purpose for local government. In Southeast China, more than 50% mangrove forests are man-made [46], most of which are dominated by viviparous species, such as *Avicennia marina* and *Kandelia obovata*. Unlike naturally occurring forests characterized by high biodiversity, these artificial forests are often mono-species or merely have several dominant species, rendering the system rather vulnerable to both internal and external disturbance. In order to enrich the biodiversity of mangrove forests and further promote the recovery of their ecosystem service, application of multi-species in mangrove reforestation is of great importance.

*Excoecaria agallocha*, known as “milk mangrove”, is an important medicinal plant and among the few non-viviparous mangrove species in Southeast China, while its restoration in nature is seldom reported. Continuous habitat loss or deterioration accompanied with little compensation has caused great challenges for survival and regeneration of non-viviparous mangrove species like *E. agallocha*. Previous researches on *E. agallocha* are mostly focused on its heredity gene and medicinal properties [47]–[50], but few can be found on its ecological adaptation to environments. Nandy et al. have ever recorded good growth of mature *E. agallocha* trees in fresh water [18], but its early response to saline conditions is still unknown. Therefore, the present study is designed to test the following three hypotheses about *E. agallocha*, expecting to fill in gaps about this species. Firstly, what is the optimal salinity for seed germination of *E. agallocha*? Our field observation shows that few *E. agallocha* seedlings can be found in forests although there are many seeds and mature *E. agallocha* trees frequently occur. Thus, we hypothesize that the saline condition in the field might be detrimental for natural regeneration of this species. Secondly, will *E. agallocha* change its salinity tolerance over time? Then, what is its tolerance limit at each developmental stage? Since juvenile stages of plants are generally more sensitive to environmental factors as compared to adult plants, we hypothesize that *E. agallocha* will increase its tolerance to salinity over time and the salinity level in its natural habitat probably approaches the talerance limit of early *E. agallocha* seedlings. Thirdly, will fertilization greatly enhance seedling growth or alleviate seedlings under stressed conditions? Since nutrient limitation is prevailing in mangrove forests, we hypothesize that nutrient addition would greatly promote growth and physiological performance of *E. agallocha* seedlings under saline conditions. To test these hypotheses, we devised a series of experiments, and the results were expected to provide instrumental information for field restoration of this species.

## Materials and Methods

### Ethics statement

No specific permits were required for the described field studies and the field studies did not involve endangered or protected species.

### Experiment for seed germination

*E. agallocha* is non-viviparous and one mature fruit contains three propagules. Mature and healthy propagules of *E. agallocha* were collected from mangrove reserve in Futian (114°1′31.57″E, 22°31′15.40″N), Shenzhen of China, which is seldom affected by human activities. The mangrove forests have average soil salinity of 15.25 psu, organic matter of 4.09%, total nitrogen of 0.139%, total phosphorus of 0.086% and total potassium of 1.25% [51]. The mature propagules were yellowish-brown in color, each with fresh weight of 30±1 mg and diameter of 4.0±0.2 mm.

Six polyethylene pots (12 cm diameter and 18 cm height) for each of the four salinity treatments were each filled with 2.5 kg coastal sand washed twice with tap water. Then, 20 propagules were sown in each pot. The pots were irrigated with artificial seawater (obtained by dissolving raw marine salt in tap water) of salinities of 0, 5, 15 and 25 psu. The water level was kept 3 to 4 mm above the sand surface and tap water was added daily to compensate evapotranspiration loss. Seawater in each pot was renewed weekly to ensure that it would not become stale. All pots were kept in an uncontrolled greenhouse under natural temperature and light. The cultivation lasted for 30 days during which air temperature was 25°C±5°C and average sunshine time was about 7.2 h/d. The seeds were considered rooted when the radicle reached 3 mm, and the unfurling of the first pair of leaves was defined as seedling establishment. The numbers of rooted seeds and established seedlings in each pot was recorded daily during the first 15 days and then every two days during the last 15 days.

### Experiment for young seedlings

Twelve polyethylene pots (21 cm diameter and 17 cm height), each containing 4 kg tap-water-washed coastal sand, were prepared. Tap water was added to each pot until the water level was 3 to 4 mm above the sand surface. Then, every three seedlings of uniform size (1.7±0.1 mm stem basal diameter and 3.5±0.2 cm stem height, at one true leaf stage) which had been cultivated under freshwater condition for 30 days from propagules were transplanted in each pot. After one week acclimatization, these seedlings were allowed to expose different salinity treatments (0, 5, 15 and 25 psu) each of which had three replicate pots. Seawater for each treatment was prepared by dissolving raw marine salt in 1.0 strength Hoagland solution [52] and was weekly renewed. Tap water was added to compensate for water loss through evapotranspiration. The treatments lasted for 120 days under the same greenhouse as above. Morphological characteristics including leaf number, maximum leaf area, stem height and stem basal diameter were recorded every 30 days.

### Experiment for old seedlings

Seedlings used for this experiment had been incubated for two years under freshwater condition. Every three uniformly sized seedlings were transplanted in each of the 24 polyethylene pots with the same size and medium as those in the young seedling experiment. Treatments began after the seedlings succeeded in colonizing the new environment (one week after transplantation). The experimental design was a completely randomized split-plot, containing eight treatments with two nutrient levels (LN: low nutrient, seawater without nutrient addition; HN: high nutrient, seawater in 1.0 strength Hoagland solution) and four salinity levels (0, 5, 15 and 25 psu). Each treatment had three replicate pots. Treatment solutions were replaced once a week, and tap water was daily added to maintain the water level of 3 to 4 mm above the sand surface. After treatments began, these seedlings were allowed to grow for 120 days under the same greenhouse as above.

Stem height and basal diameter, leaf number and maximum leaf area were recorded every 30 days. After 120 days treatment, all seedlings were harvested, rinsed thoroughly with deionized water, dried at 105°C to constant weight for the measurements of the biomass and its partition (shoot to root biomass ratio, S/R).

Relative growth rates (RGRs) were estimated. Before treatments began, 20 transplanted seedlings were randomly selected to measure stem height (*H*), stem basal diameter (*D*) and total biomass (*B*). According to the logarithmic relation between *B* and *D* ^2^ *H*, the equation was simulated as following:
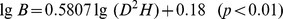
The initial total biomass of each seedling was then estimated using the above equation and RGRs were calculated as:
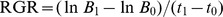
Here, *B_0_* and *B_1_* are total biomass at the beginning (*t_0_*) and end (*t_1_*) of the experimental period [53].

All physiological analyses were done during the last week of the cultivation. For each seedling, 3 to 4 fully expanded leaves were selected to measure net photosynthetic rate (*P_n_*), transpiration rate (*T*_r_) and stomatal conductance (*g*_s_) by using a portable photosynthesis system (LI-6400XT, U.S.). Gas exchange measurements were conducted between 10 a.m. and 11 a.m. with ambient CO_2_ concentration of 387±2 μmol ml^−1^, illumination intensity of 1000 μmol m^−2^ s^−1^, air temperature of 32.4±0.8°C and relative humidity of 63%±3%. Water use efficiency (WUE) was calculated through dividing *P_n_* by *T*_r_. These measured leaves were sampled for further chemical analyses.

Activities of catalase (CAT) and superoxide dismutase (SOD) as well as malondialdehyde (MDA) content were determined according to those described by Aebi [54] and Ye et al. [55]. Salt content in leaf tissues was measured following the method by Ye et al. [55] with some modification. One leaf was ground in double distilled water. The homogenate was centrifuged at 10000×*g* for 3 min and the conductivity of the supernatant was measured with a conductivity meter made by Orion. Conductivity values were then converted to salt contents and the final salt content (%) in leaf tissue solution was expressed as salt per gram of water in leaf tissue. Proline contents in leaf tissues were determined according to the method described by Bates et al. [56], using an extract of 0.3 g fresh leaf materials in aqueous sulphosalicylic acid. The extracted proline was reacted with ninhydrin to form a chromophore and then the absorbance at 520 nm was measured for final determination of proline content.

### Statistical analysis

All values were expressed as mean ± standard deviation (S.D.) values of 6 (germination experiment) or 3 (seedling experiments) replicates. For either young or old seedlings cultivated with 1.0 strength Hoagland solution, differences in parameters obtained in each measurement event among four salinity treatments (0, 5, 15 and 25 psu) were analyzed by 1-way ANOVA. For old seedlings, differences among salinity treatments and between nutrient levels, and interactions of these two factors were analyzed by 2-way ANOVA. If any significant difference was found, the Student-Newman-Keuls multiple comparison method was involved.

## Results

### Responses to salinity of *E. agallocha* at different developmental stages

**Figure 1:**
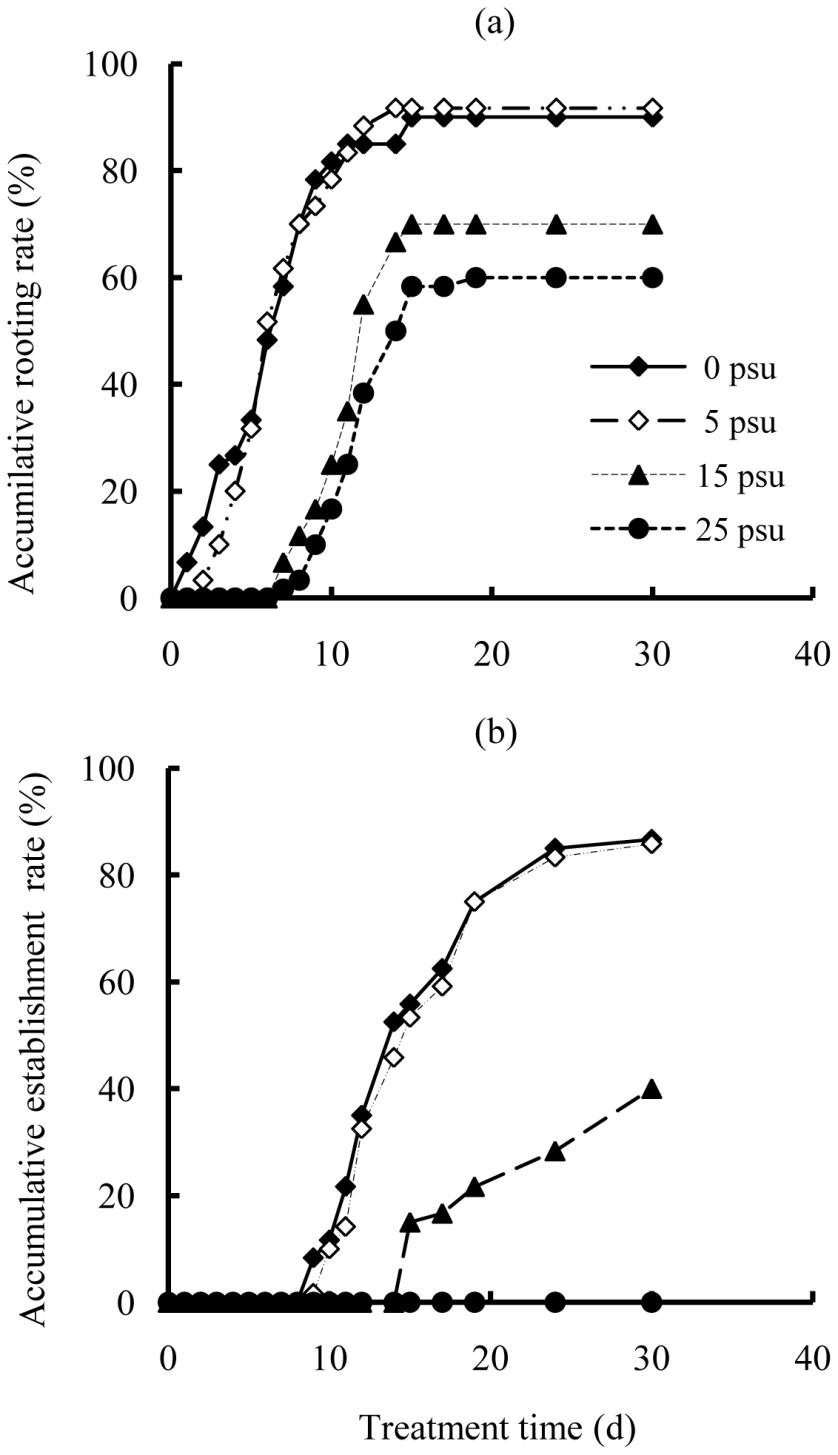
Effects of salinity on seed germination of *E. agallocha*.

At 0 and 5 psu, propagules of *E. agallocha* began to emerge roots 1–2 d after being sowed (Fig. 1a). Root emergence continued for a maximum period of 15 and 14 d at 0 and 5 psu, and the final rooting rates were both over 90%. Compared with 0 or 5 psu treatments, salinity of 15 and 25 psu delayed root initiation by several days. Root emergence at 15 and 25 psu was firstly recorded on the 6th day, with final rooting rates of 70.0±1.2% and 60.0±2.3%, respectively. Propagules of *E. agallocha* had similar seedling establishment between 0 and 5 psu, with first pair leaves unfurled 9 days after sowing and final establishment rates over 85%. (Fig. 1b). Compared with low salinities, increasing salinity level to 15 psu postponed seedling establishment for 6 days and the final establishment rate decreased to 37.5%. At 25 psu, all seeds failed to establish though some of them had initiated roots.

**Figure 2:**
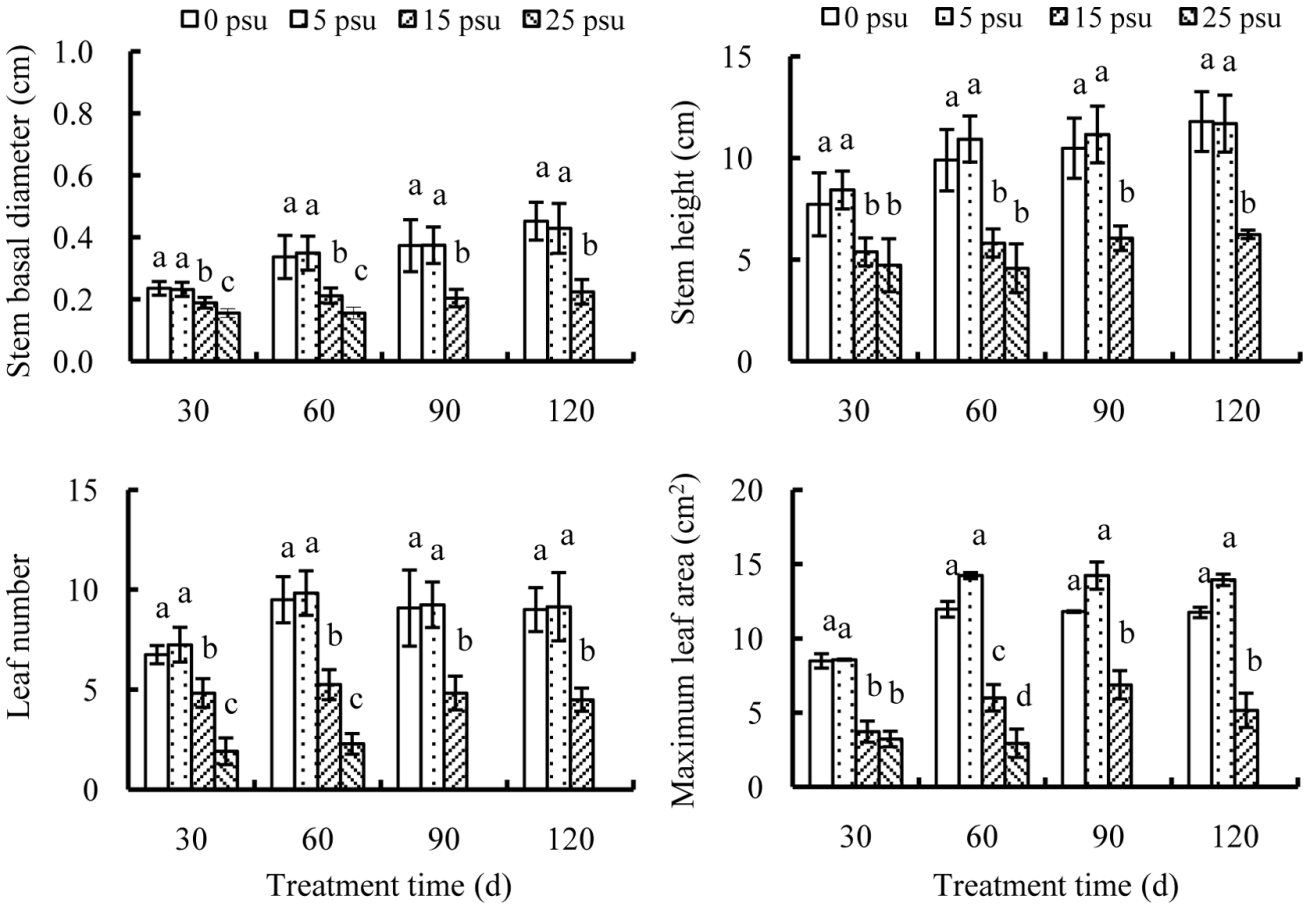
Effects of salinity on growth of young seedlings. Growth parameters include stem basal diameter, stem height, leaf number and maximum leaf area of young *E. agallocha* seedlings cultivated with 1.0 strength Hoagland solution. Mean and SD of three replicates are shown, and means at the same salinity with different letters are significantly different at 0.05 according to 1-way ANOVA test.

In general, growth performance of young seedlings was favorable at salinity of 0 and 5 psu, followed by 15 psu and 25 psu (Fig. 2). Below 15 psu treatments, all seedlings survived throughout the 120 days' cultivation, whereas at 25 psu, plant deaths occurred after 60 days and all seedlings died within 30 days. Dynamics of all the measured morphologic parameters showed similar tendency at 0 and 5 psu. The stem basal diameter and stem height of seedlings at 0 or 5 psu increased gradually with time, while leaf number and leaf area increased considerably during the first 60 days and then changed little. Elevating salinity to 15 psu evidently compromised growth performance. Stem basal diameter and stem height increased little with time and leaf number even declined, whereas the maximum leaf area increased during the first 90 days and decreased afterwards. At 25 psu, all seedlings failed to survive the whole experiment period.

**Figure 3:**
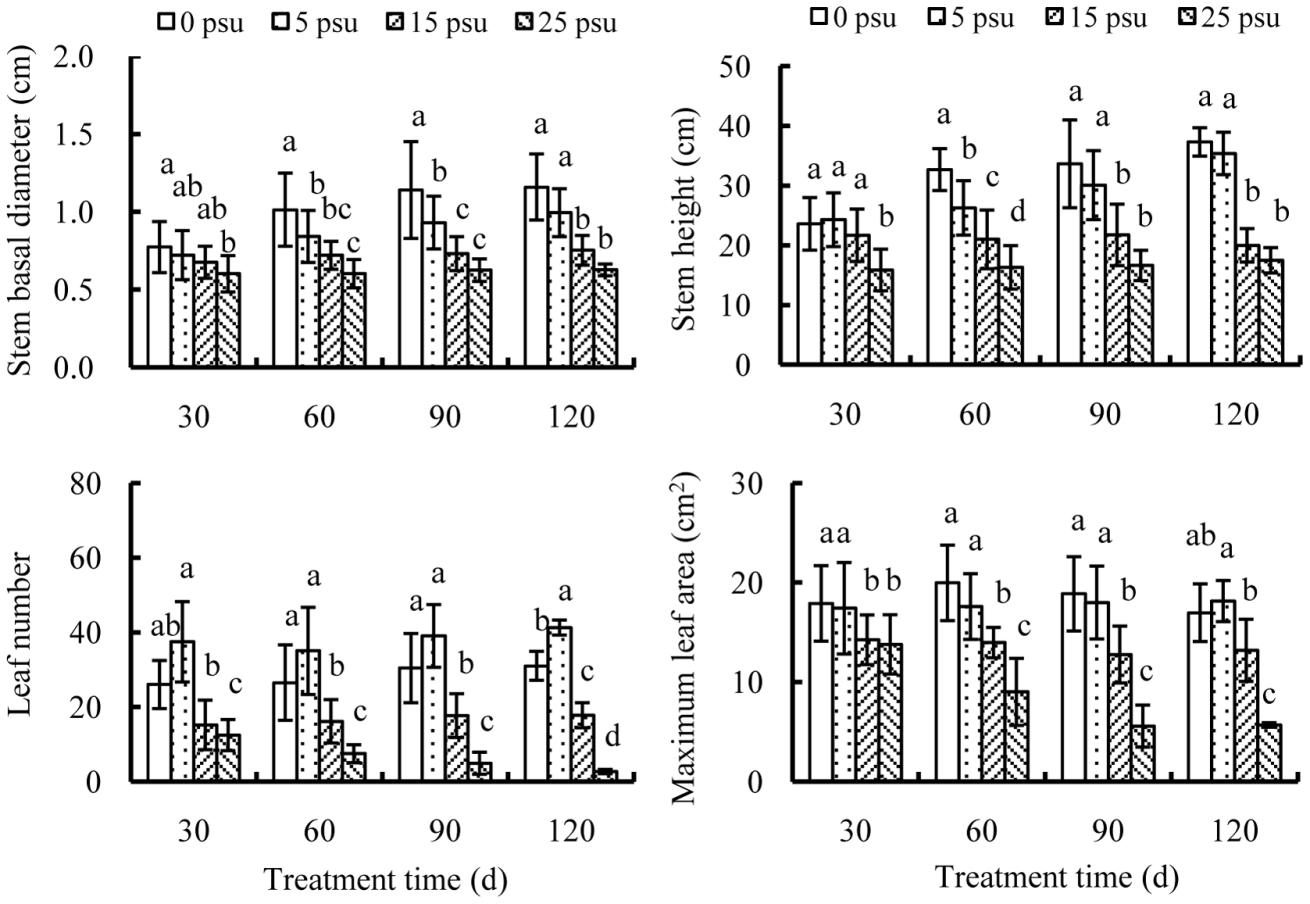
Effects of salinity on growth of old seedlings. Growth parameters include stem basal diameter, stem height, leaf number and maximum leaf area of old *E. agallocha* seedlings cultivated with 1.0 strength Hoagland solution. Mean and SD of three replicates are shown, and means at the same salinity with different letters are significantly different at 0.05 according to 1-way ANOVA test.

Old seedlings were different from young seedlings in response to salinity. All seedlings survived the whole experiment period. Growth performance declined in the order of 5 psu ≥0 psu >15 psu >25 psu (Fig. 3). Similar to young seedlings, at 0 and 5 psu, stem basal diameter, stem height and leaf number increased gradually over time, while increases in maximum leaf area were less visible. At the end of the cultivation, all growth parameters except leaf number showed no differences between 0 and 5 psu treatment. Patterns of stem basal diameter and stem height were similar between 15 and 25 psu, with no significant changes over time. At 15 psu, leaf number increased a little while maximum leaf area declined with time. At 25 psu, older leaves gradually detached from seedlings, but few new leaves were produced during the experiment, resulting in evident declines in leaf number and maximum leaf area.

### Combined effects of salinity and nutrient on old seedlings of *E. agallocha*

**Figure 4:**
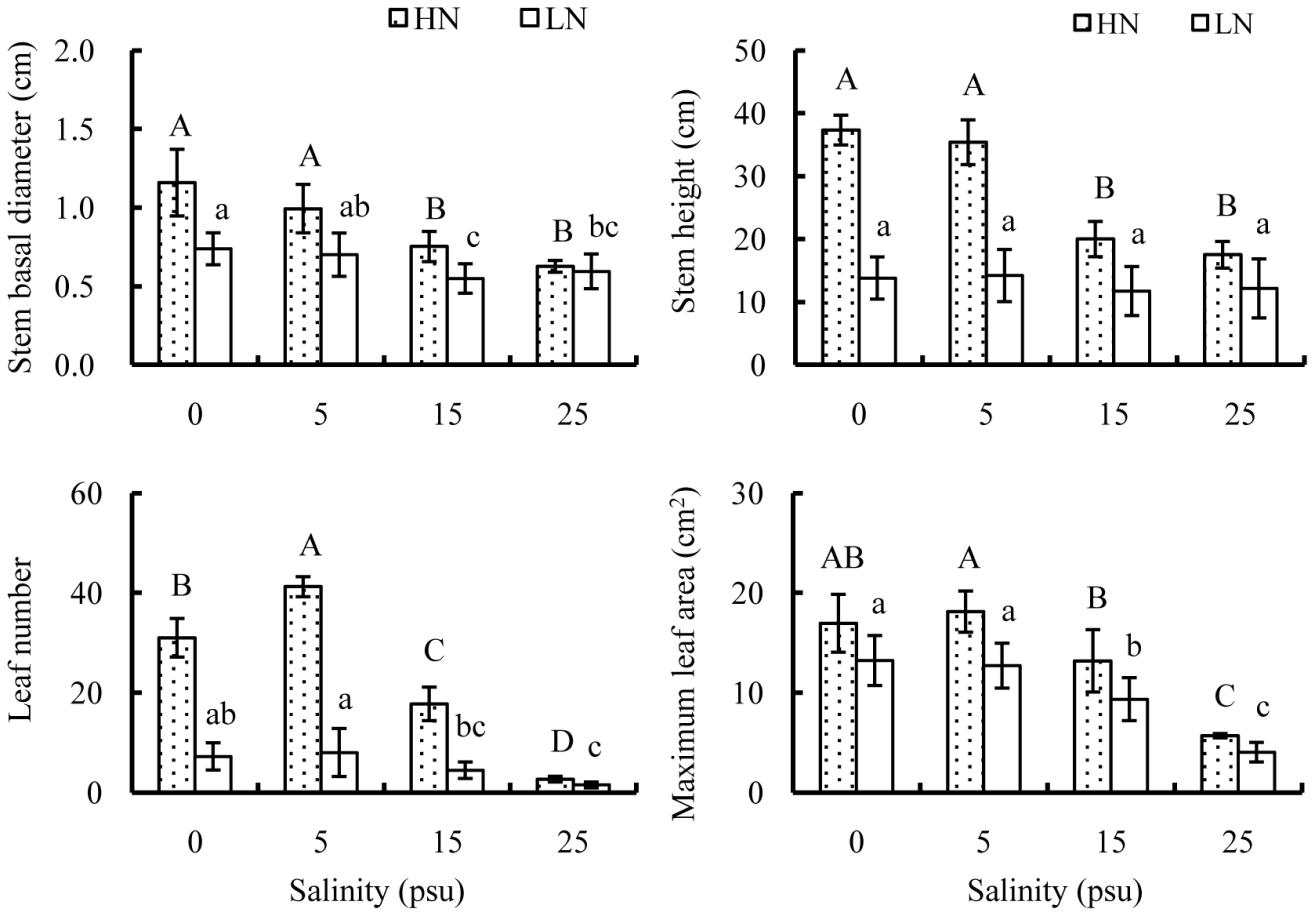
Effects of salinity and nutrient on growth of old seedlings. Growth parameters of stem basal diameter, stem height, leaf number and maximum leaf area were measured after 120 days cultivation. Mean and SD of three replicates are shown, and means at the same nutrient level with different letters are significantly different at 0.05 according to 1-way ANOVA test. HN: high nutrient, fertilized with 1.0 strength Hoagland nutrient solution; LN: low nutrient, unfertilized.

Both salinity and nutrient had significant effects on morphologic parameters of old seedlings after 120 days cultivation, and their interactive effects on stem basal diameter, stem height and leaf number were very significant (Table 1). At any salinity, seedling growth was more vigorous at high than low nutrient, while the differences in morphologic parameters between the two nutrient levels gradually decreased as salinity increased (Fig. 4). At high nutrient, stem basal diameter and stem height varied little either between salinity of 0 and 5 psu or between 15 and 25 psu, while the values at salinities above 15 psu were significantly lower than those at salinities below 5 psu. The maximum value of leaf number occurred at 5 psu, about 1.3, 2.3 and 15.5 times those at 0, 15 and 25 psu, respectively. Similarly, maximum leaf area was highest at 5 psu, not significantly different from that at 0 psu, but 37.5% and 219.0% higher than those at 15 and 25 psu, respectively. Decreasing nutrient availability reduced the differences in stem basal diameter, stem height and leaf number among salinities. Similar to those at high nutrient, decreases in stem basal diameter, leaf number and maximum leaf area at low nutrient were significant with increasing salinity, while significant variations in stem height were not detected among salinities.

**Table 1:** Results of 2-way ANOVA on growth and physiological parameters of old *E. agallocha* seedlings showing interactions between salinity and nutrient.

Parameter	Factor		
	Salinity	Nutrient	Salinity × Nutrient
Stem basal diameter	20.284***	45.784***	4.568**
Stem height	28.050***	172.901***	17.173***
Leaf number	62.729***	215.333***	27.049***
Maximum leaf area	35.792***	24.819***	1.031
Total biomass	5.497**	12.453**	1.608
S/R	5.249**	17.128***	0.407
RGR	71.920***	54.150***	12.003***
*P_n_*	135.704***	211.834***	19.325***
*T_r_*	109.575***	274.099***	46.485***
*g_s_*	140.511***	490.896***	85.538***
WUE	29.155***	16.489**	6.076**
Salt content	25.311***	0.501	1.162
Proline	16.828***	39.328***	3.206
MDA	29.697***	25.805**	2.633
CAT	2.821	926.322***	1.401
SOD	3.450*	4.047	2.532

*F*-values are given and significant effects are denoted as: *0.01<*P*<0.05, **0.001<*P*<0.01, ***P<0.001.

**Figure 5:**
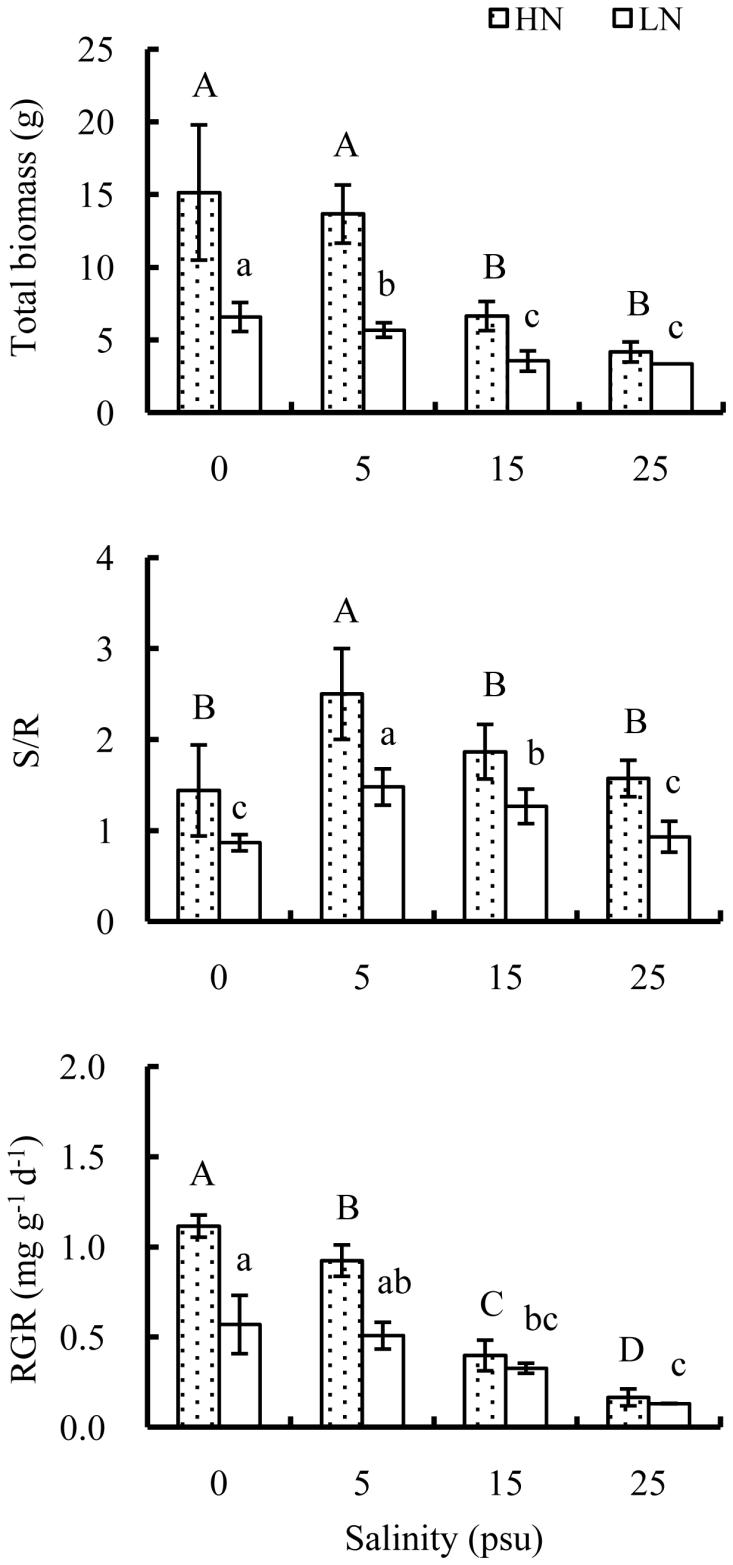
Effects of salinity and nutrient on biomass partition and RGR of old seedlings. Parameters of total biomass, S/R and RGR were measured after 120 days cultivation. Mean and SD of three replicates are shown, and means at the same nutrient level with different letters are significantly different at 0.05 according to 1-way ANOVA test. HN: high nutrient, fertilized with 1.0 strength Hoagland nutrient solution; LN: low nutrient, unfertilized.

Salinities above 15 psu significantly depressed biomass accumulation of old *E. agallocha* seedlings, and the inhibitory effects of high salinity became more pronounced when coupled with low nutrient (Fig. 5). No significant interaction between salinity and nutrient was detected on total biomass (Table 1), while the value of total biomass at low nutrient only accounted for 43.5%, 41.6%, 53.5% and 83.5% of those at high nutrient at 0, 5, 15 and 25 psu, respectively. Similarly, in spite of significant higher values of S/R at high than low nutrient, patterns of S/R with salinity were similar between the two nutrient levels: significantly increasing with salinity increasing from 0 to 5 psu and then decreasing sharply with further increasing salinity from 5 to 25 psu. High salinity or low nutrient significantly compromised RGR of *E. agallocha* seedlings, while the decline trend in RGR with salinity was more pronounced at high than low nutrient.

**Figure 6:**
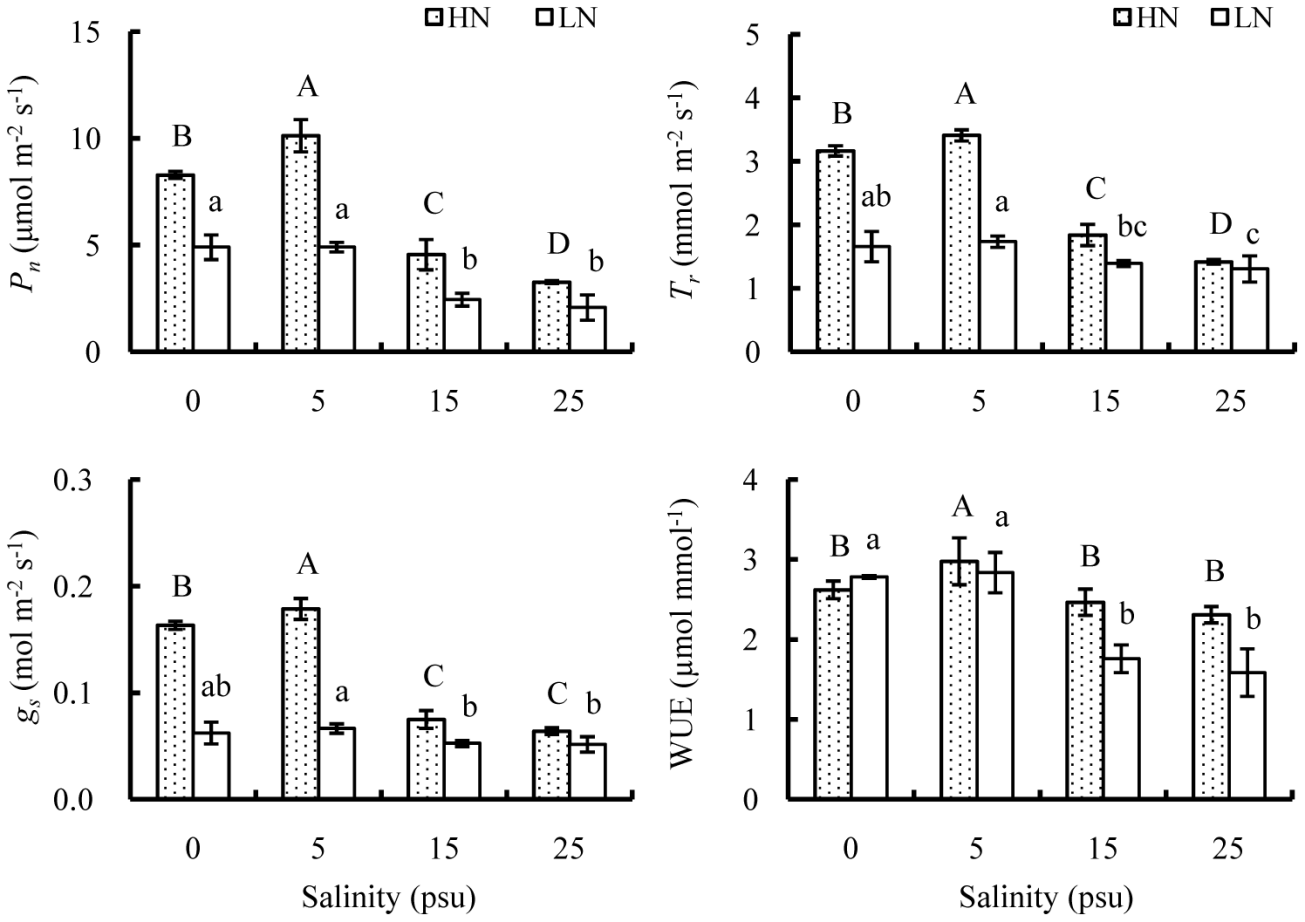
Effects of salinity and nutrient on leaf gas exchange of old seedlings. Physiological parameters include *P_n_*, *T_r_*, *g_s_* and WUE. Mean and SD of three replicates are shown, and means at the same nutrient level with different letters are significantly different at 0.05 according to 1-way ANOVA test. HN: high nutrient, fertilized with 1.0 strength Hoagland nutrient solution; LN: low nutrient, unfertilized.

Effects of salinity were significant on gas exchange of *E. agallocha* leaves (Fig. 6). Gas exchange was also significantly enhanced by high nutrient, as shown by significantly increasing *P_n_*, *T_r_*, *g_s_* and WUE at high compared to low nutrient. However, differences in *P_n_*, *T_r_* and *g_s_* among the four salinity treatments seemed more pronounced at high than low nutrient. At high nutrient, gas exchange was greatly promoted by elevating salinity level from 0 to 5 psu, but significantly depressed as salinity increased to 15 psu. As increasing salinities from 15 to 25 psu, *P_n_* and *T_r_* continued to decline while *g_s_* and WUE no more significantly changed. Differently, at low nutrient, gas exchange differed little between 0 and 5 psu salinities, but significantly inhibited at 15 psu, while gas exchange at 25 psu was comparable to that at 15 psu.

**Figure 7:**
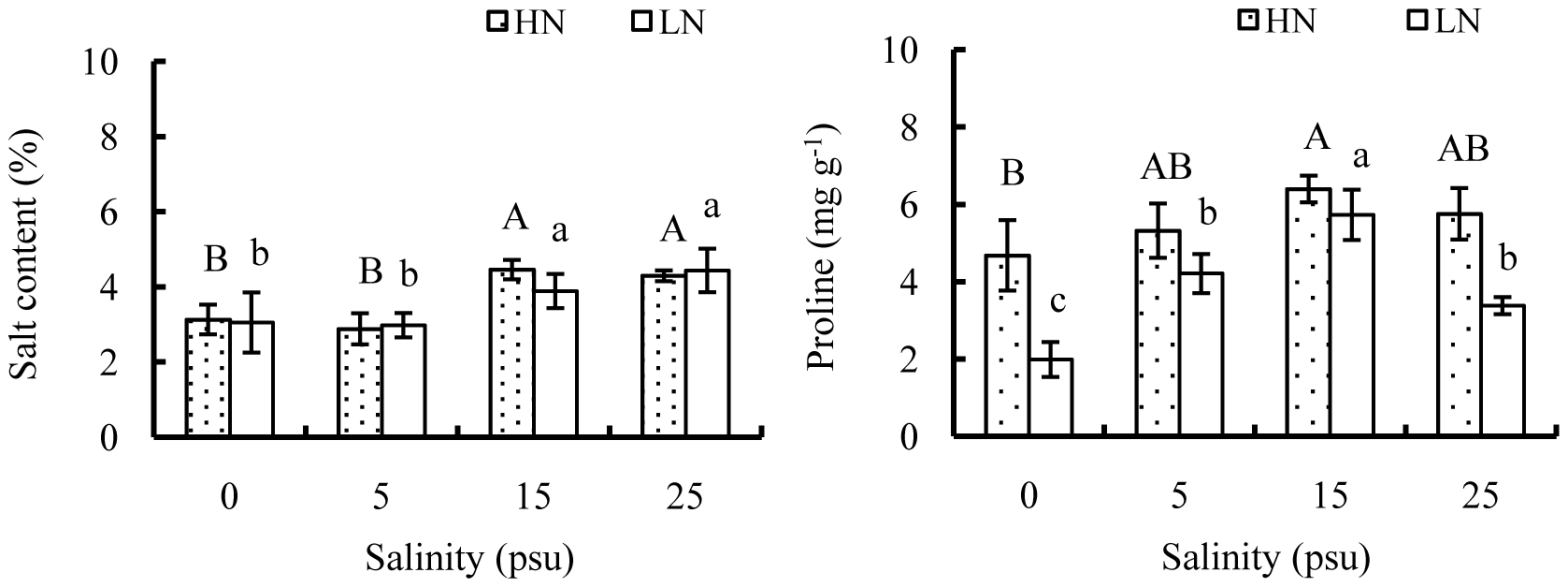
Effects of salinity and nutrient on salt content and proline accumulation of old seedlings. Mean and SD of three replicates are shown, and means at the same nutrient level with different letters are significantly different at 0.05 according to 1-way ANOVA test. HN: high nutrient, fertilized with 1.0 strength Hoagland nutrient solution; LN: low nutrient, unfertilized.

Salt contents in *E. agallocha* leaves were significantly affected by salinity but not by nutrient level, and there were no interactive effects between salinity and nutrient level on this parameter (Table 1). Increasing salinity from 0 to 5 psu had no significant effect on leaf salt contents, but elevating salinity level from 5 to 15 increased the values by 55.0% and 30.6% at high and low nutrient levels, respectively (Fig. 7). Salt contents varied little with further increasing salinity from 15 to 25 psu. Proline contents were significantly enhanced by high nutrient (Table 1, Fig. 7), with the values at high nutrient about 2.4, 1.3, 1.1 and 1.7 times those at low nutrient at salinity of 0, 5, 15 and 25 psu, respectively. However, there were no consistent responses in proline contents to salinity. Elevating salinity level from 0 to 15 psu greatly accelerated proline accumulation in leaves, but further increasing salinity from 15 to 25 psu imposed an opposite effect on proline accumulation.

**Figure 8:**
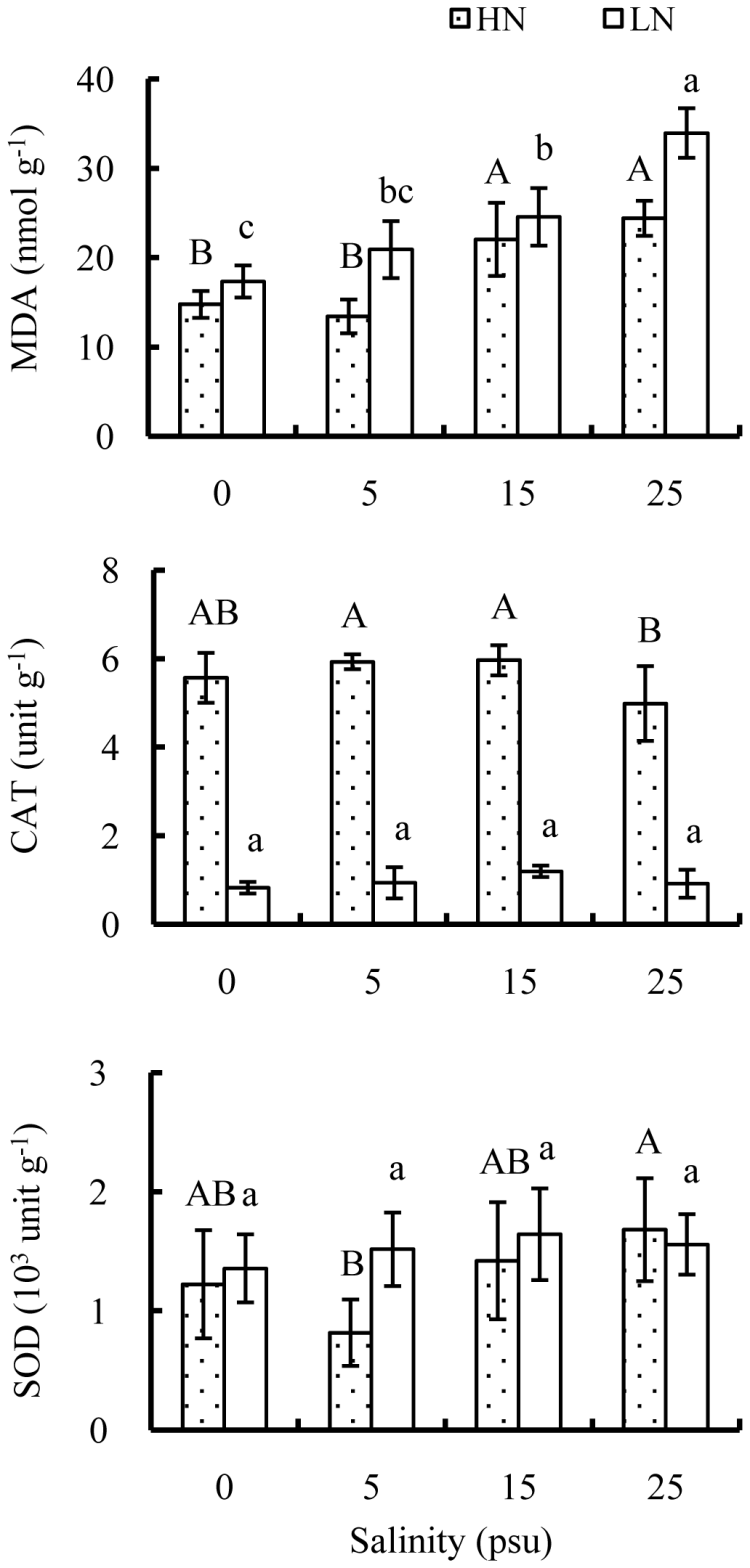
Effects of salinity and nutrient on MDA contents, CAT and SOD activities of old seedlings. Mean and SD of three replicates are shown, and means at the same nutrient level with different letters are significantly different at 0.05 according to 1-way ANOVA test. HN: high nutrient, fertilized with 1.0 strength Hoagland nutrient solution; LN: low nutrient, unfertilized.

MDA contents in leaves were evidently promoted either by elevating salinity level or decreasing nutrient level (Table 1). High nutrient decreased MDA contents by 14.8%, 35.8%, 10.3% and 28.1% at 0, 5, 15 and 25 psu, respectively (Fig. 8). There were no significant effects of salinity on CAT activity, but CAT activity was steeply stimulated by nutrient addition, with values at high nutrient about 6.7, 6.3, 5.0 and 5.4 times those at low nutrient at salinity of 0, 5, 15 and 25 psu, respectively. In contrast, SOD activity showed no significant differences between nutrient levels, but significantly activated by increasing salinity from 5 to 25 psu at high nutrient.

## Discussion

### Effects of salinity at different developmental stages

Seed germination is extensively reported to be reduced, retarded [4], [57], [58] or completely inhibited [59] at high salinities. Similarly, seeds of *Excoecaria agallocha* showed clear preference for low salinities from 0 to 5 psu, and salinity beyond 15 psu significantly delayed root initiation and seedling establishment. High salinities could inhibit seed germination either by impeding water uptake [60] or through facilitating the intake of toxic ions [61], [62]. When compared to seed rooting, effects of high salinities seemed more severe on seedling establishment. Salinity of 25 psu was deadly to *E. agallocha* seeds, under which no seedlings succeeded to establish. Likewise, root initiation of *Ae. corniculatum* was not evidently depressed by increasing salinity, but the final establishment percentages decreased significantly at salinities over 25 psu [6]. One possible explanation is that when departing from mother trees, seeds often store a certain amount of water to help go through water deficit during early germination; when further development gradually depletes internal water storage, while water uptake from external environment is inhibited by high salinity, seedlings would be confronted with severe physiological drought, leading to the failure of establishment.

Although mangroves can survive under a wide range of salinity, their growth, stature and productivity varied dramatically along salinity gradients [1]–[3]. Salinity affects both leaf initiation and leaf area expansion [7], [63], and high salinities generally result in dwarfed plants with small and thick leaves [4], [19]. Similar to mature *E. agallocha* trees [18], young *E. agallocha* seedlings had vigorous growth at low salinity of 0 and 5 psu, with stems elongated and basal diameter expanded over time. Accelerated senescence of old leaves coupled with decreases in leaf initiation at 15 psu resulted in negative leaf gain over time, implying that 15 psu approaches the limit of salt tolerance for young *E. agallocha* seedlings. Salinity beyond 25 psu was lethal to young seedlings since all plants died within 90 days.

Sensitivity of plants to salinity depends on their developmental stages. As compared to young seedlings of *E. agallocha*, old seedlings were more tolerant to high salinities. Although old seedlings were severely retarded at 15 psu with small and dark leaves, the stressed plants were healthy in appearance with no wilting symptoms observed. In addition, these seedlings slowly but continually produced new leaves, leading to a slight increase in leaf number over time, which implied that old seedlings of *E. agallocha* can tolerate salinity up to 15 psu. High salinity or prolonged salt stress can accelerate leaf mortality and depress leaf production, frequently leading to plant death in the long term [11], [64], [65]. Likewise, accelerated leaf senescence and mortality of *E. agallocha* seedlings at 25 psu was accompanied by little leaf production, resulting in negative foliage gain over time. At the end of the experiment, the leaf number approximately declined to zero, suggesting that although having lived through the whole experiment period, old seedlings of *E. agallocha* cannot survive salinities above 25 psu in the long run.

### Combined effects of salinity and nutrient

At either nutrient level, seedling growths were most vigorous at salinities of 0 to 5 psu, and increasing salinity beyond 15 psu significantly depressed seedling growth. Fewer leaves along with smaller leaf area may be adaptations to salt stress, expecting to reduce water loss through evapotranspiration. Similar to the observation by Ball and Sobrado [66], nutrient addition to some extent alleviated salt-induced reduction of seedling growth. Salt tolerance often involves the synthesis and accumulation of N-based compounds which act in osmotic adjustment [41], [67], [68]. At high nutrient level, the improved salt tolerance of *E. agallocha* seedlings might be attributed to enhanced osmoregulation by additional nitrogen supply.

Nutrient addition promoted shoot growth and increased branch number of *Av. germinans* [33], enhanced leaf production and expansion of *Av. marina* [32], [34] and promoted stem elongation of dwarfed mangroves [38]. Likewise, adding nutrient induced vigorous growth of *E. agallocha*, especially at low salinities. However, promoting effects of nutrient addition was compromised at high salinities, as shown by narrowed differences in morphologic parameters between the two nutrient levels as salinities increased from 5 to 25 psu. Similar results were also attained for other mangrove species like *K. candel* [17] and *Av. marina* [26]. When grown at favorable salinities, seedling growth might be primarily limited by nutrient availability, thus, adding nutrient can greatly enhance growth performance. On the contrary, when adverse saline conditions became the dominate factor over nutrient limit to depress seedling growths, high cost of osmotic adjustment [17] and ion toxicity [4], [69] might prevent plants from taking advantage of improved nutrient supply. High contents of Na^+^ in soils typically reduce the uptake of K^+^ [25], [70] and other nutrients like Ca^2+^ and Mg^2+^ [4], leading to ion imbalance in leaves [63], [71], while the presence of excessive Cl^−^ salts could inhibit NO_3_^−^ uptake [63], which interrupts protein synthesis mechanism [72].

In addition, high salinity and low nutrient significantly decreased biomass accumulation and RGR, and changed biomass partitioning of *E. agallocha* seedlings. Similar observation was also recorded in mangrove species of *Av. marina* and *B. parviflora* [4], [63], *K. candel* [17] and *S. lanceolata* [9]. Reduction of leaf expansion rates was reported to be one main reason for growth decrease under salt stress [73]. In this study, decreases in total biomass and RGRs were accompanied by decreases in both leaf number and area. As salinity increased from 5 to 25 psu or as nutrient level decreased, a shift in biomass allocation from shoots to roots of *E. agallocha* seedlings occurred. One possible explanation might be that increasing biomass investments to roots could facilitate water and nutrient uptake under high salinities. In the same way, seedlings at low nutrient enhanced root allocation in order for nutrient acquisition.

Decreases in stomatal conductance (*g_s_*) with increasing salinity are typical responses of mangroves [19], [63]. Stomatal closure at high salinities minimizes water loss through transpiration, which is however, accompanied by reduced carbon gain, leading to suppression of photosynthesis [18], [25], [74], [75]. In the present study, net photosynthetic rate (*P_n_*) and transpiration rate (*T_r_*) of *E. agallocha* seedlings decreased in parallel with *g_s_* as salinity increased from 5 to 25 psu. Moreover, in spite of a sharp decrease in *T_r_* through stomatal regulation, *E. agallocha* seedlings failed to maintain high water use efficiency (WUE) at elevated salinities. Dramatic decreases in WUE coupled with significantly lowered *P_n_* possibly accounted for the poor growth performance at salinities above 15 psu. In addition to stomatal limitation, nutrient imbalance might be another possible reason for decreased photosynthesis at high salinities, since salt induced ion imbalance, especially K and Mg deficiency, was extensively reported to suppress chlorophyll synthesis and photosynthetic capacity [18], [70]. On the contrary, nutrient addition significantly enhanced *P_n_*, *T_r_*, *g_s_* as well as WUE of *E. agallocha* seedlings, similar observation was also recorded on *K. candel* seedlings [17] and dwarfed *R. mangle* trees [38]. Furthermore, gas exchange in terms of *P_n_*, *T_r_* and *g_s_* was interactively affected by nutrient and salinity, which is in conformity with growth responses in morphological parameters and RGR. Although *P_n_*, *T_r_* and *g_s_* were promoted by high nutrient at any salinity, the differences in these parameters between nutrient levels decreased with increasing salinity.

When in salt stressed habitat, mangroves could accumulate inorganic ions or compatible solutes like betaine, proline, or sugar alcohol as osmotica to counter the toxic effects of salinity [6], [21], [76]. In this study, increasing salinities beyond 15 psu induced evident increases in salt content of *E. agallocha* seedlings, while nutrient addition had no significant effect on salt contents. Differently, proline contents increased significantly with salinity increasing from 0 to 15 psu, but decreased as it increased to 25 psu. Flowers et al. [41] indicated that the accumulation of proline is strongly affected by nitrogen availability, and the inhibition of nitrogen uptake at high salinities may be the reason for decreased proline contents. In the same way, high nutrient significantly enhanced proline accumulation in *E. agallocha* seedlings, possibly contributing to osmotic adjustment.

An inevitable consequence of salt stress is the excessive generation of reactive oxygen species which can severely permeabilize membranes by causing lipid peroxidation [16], [77], [78]. MDA, an effective indicator of oxidation damage, accumulated considerably with increasing salinities beyond 15 psu, and its contents were significantly higher at low than high nutrient level, which suggests that lipid peroxidation was aggravated under salt stress, while nutrient addition could greatly alleviate oxidation damage. In addition, antioxidative enzymes like CAT, SOD and peroxidase are often triggered under stressed conditions to defend against oxidation damage [23], [79], [80]. SOD activities of *E. agallocha* seedlings were stimulated by increasing salinities from 5 to 25 psu, especially at high nutrient. However, CAT was not activated by salt stress, but it was greatly promoted by increasing nutrient availability, which possibly explained the significantly lowered MDA contents at high nutrient.

## Conclusion

*E. agallocha* is highly sensitive to salinity, especially at early developmental stages. The favorable salinity range for seed germination is below 5 psu, and salinity of 15 psu decreased seedling establishment rate to 37%, which partly explained why few seedlings can be found in the mangrove reserve which has soil salinity slightly higher than 15 psu. The adverse saline condition in the field might act as a primary obstacle for natural regeneration of this species. Thus, artificial breeding and culture should be adopted to ensure higher survival rate of *E. agallocha* seedlings.

Then when to replant this species in the field? Results from the present study indicate that seedlings as young as one-month old can hardly tolerant salinity above 15 psu, while for two-year old seedlings, they can adapt to salinity up to 15 psu although failing to surviving 25 psu salinity in the long run, which indicates that *E. agallocha* increased salinity tolerance over time and two-year old seedlings could be chosen for *E. agallocha* restoration in sites where soil salinity is up to 15 psu. In addition to salinity, nutritional limitation prevailing in mangrove forests is another challenge for successful reforestation. The current study shows that nutrient addition could not only greatly enhance plant growth, but also alleviate salt-induced damage to plant physiology. Therefore, applying nutrient rationally could be very helpful in promoting survival of *E. agallocha* seedlings in plantations with relatively high salinity.

On the other hand, we should be aware that due to the particularity of their natural habitat, field restoration of mangrove forests is confronted with many other challenges. Duration and frequency of seawater inundation, predation by crabs and gastropods, soil physiochemical properties, inter- and intra-species competition, and anthropogenic disturbance might be equally important in determining whether *E. agallocha* seedlings would sustainably develop in nature. And researches on these environmental factors as well as various other mangrove species are under way in order for the prosperity of mangrove ecosystems.

## References

[1] Lovelock CE, Feller IC, Mckee KL, Engelbrecht BMJ, Ball MC (2004) The effect of nutrient enrichment on growth, photosynthesis and hydraulic conductance of dwarf mangroves in Panamá. Funct Ecol 18: 25–33.

[2] McKee KL, Feller IC, Popp M, Wanek W (2002) Mangrove isotopic (δ^15^N and δ^13^C) fractionation across a nitrogen vs. phosphorus limitation gradient. Ecology 83: 1065–1075.

[3] Naidoo G (2006) Factors contributing to dwarfing in the mangrove *Avicennia marina* . Ann Bot-London 97: 1095–1101. 10.1093/aob/mcl064 PMC2803391 16565149

[4] Patel NT, Gupta A, Pandey AN (2010) Salinity tolerance of *Avicennia marina* (Forssk.) Vierh. from Gujarat coasts of India. Aquat Bot 93: 9–16.

[5] Ye Y, Lu CY, Wong YS, Tam NFY (2004) Diaspore Traits and Inter-tidal Zonation of Non-viviparous Mangrove Species. Acta Botanica Sinica 46(8): 896–906.

[6] Ye Y, Tam NFY, Lu CY, Wong YS (2005) Effects of salinity on germination, seedling growth and physiology of three salt-secreting mangrove species. Aquat Bot 83: 193–205.

[7] Clough BF (1984) Growth and salt balance of the mangrove *Avicennia marina* (Forsk.) Vierh. and *Rhizophora stylosa* Griff. in relation to salinity. Aust J Plant Physiol 11: 419–430.

[8] Ball MC, Anderson JM (1986) Sensitivity of photosystem II to NaCl in relation to salinity tolerance: comparative studies with thylakoids of the salt-tolerant mangrove, *Avicennia marina*, and the salt-sensitive pea, *Pisum sativum* . Aust J Plant Physiol 13: 689–698.

[9] Ball MC, Pidsley SM (1995) Growth responses to salinity in relation to distribution of two mangrove species, *Sonneratia alba* and *S. lanceolata*, in northern Australia. Funct Ecol 9: 77–85.

[10] Medina E, Francisco M (1997) Osmolality and δ^13^C of leaf tissue of mangrove species from environments of contrasting rainfall and salinity. Estuar Coast Shelf S 45: 337–344.

[11] Munns R, Termaat A (1986) Whole-plant responses to salinity. Aust J Plant Physiol 13: 143–160.

[12] Suarez N, Medina E (2005) Salinity effect on plant growth and leaf demography of the mangrove, *Avicennia germinans* L. Trees 19: 721–727.

[13] Tanaka Y, Hibino T, Hayashi Y, Tanaka A, Kishitani S, et al (1999) Salt tolerance of transgenic rice over-expressing yeast mitochondrial Mn-SOD in chloroplasts. Plant Sci 148: 131–138.

[14] Li N, Chen SL, Zhou XY, Li CY, Shao J, et al (2008) Effect of NaCl on photosynthesis, salt accumulation and ion compartmentation in two mangrove species, *Kandelia candel* and *Bruguiera gymnorrhiza* . Aquat Bot 88: 303–310.

[15] Khan MA, Ungar IA, Showalter AM (2000) The effect of salinity on the growth, water status, and ion content of a leaf succulent perennial halophyte, *Suaeda fruiticosa* (L.) Forssk. J Arid Environ 45: 73–84.

[16] Hasegawa PM, Bressan RA, Zhu JK, Bohnert HJ (2000) Plant cellular and molecular responses to high salinity. Annu Rev Plant Physiol Plant Mol Biol 51: 463–499. 15012199 10.1146/annurev.arplant.51.1.463

[17] Kao WY, Tsai HC, Tsai TT (2001) Effect of NaCl and nitrogen availability on growth and photosynthesis of seedlings of a mangrove species, *Kandelia candel* (L.) Druce. J Plant Physiol 158: 841–846.

[18] Nandy P, Das S, Ghose M, Spooner-Hart R (2007) Effects of salinity on photosynthesis, leaf anatomy, ion accumulation and photosynthetic nitrogen use efficiency in five Indian mangroves. Wetl Ecol Manag 15: 247–357.

[19] Naidoo G, Hiralal O, Naidoo Y (2011) Hypersalinity effects on leaf ultrastructure and physiology in the mangrove *Avicennia marina* . Flora 206: 814–820.

[20] Cheng H, Wang YS, Ye ZH, Chen DT, Wang YT, et al (2012) Influence of N deficiency and salinity on metal (Pb, Zn and Cu) accumulation and tolerance by *Rhizophora stylosa* in relation to root anatomy and permeability. Environ Pollut 164: 110–117. 22361050 10.1016/j.envpol.2012.01.034

[21] Dasgupta N, Nandy P, Sengupta C, Das S (2012) Protein and enzymes regulations towards salt tolerance of some Indian mangroves in relation to adaptation. Trees 26: 377–391.

[22] Nandy P, Dasgupta N, Das S (2009) Differential expression of physiological and biochemical defense mechanisms of some Indian mangroves towards salt tolerance. Physiol Mol Biol Plants 151(2): 151–160. 10.1007/s12298-009-0017-7 PMC3550367 23572924

[23] Dasgupta N, Nandy P, Tiwari C, Das S (2010) Salinity imposed changes of some isozymes and total leaf protein expression in five mangroves from two different habitats. J Plant Interact 5(3): 211–221.

[24] Prochazkova D, Sairam RK, Srivastava GC, Singh DV (2001) Oxidative stress and antioxidant activity as the basis of senescence in maize leaves. Plant Sci 161(4): 765–771.

[25] Ball MC, Farquhar GD (1984) Photosynthetic and stomatal responses of two mangrove species, *Aegiceras corniculatum* and *Avicennia marina*, to long term salinity and humidity conditions. Plant Physiol 74: 1–6. 16663359 10.1104/pp.74.1.1 PMC1066613

[26] Naidoo G (1987) Effects of salinity and nitrogen on growth and plant water relations in the mangrove *Avicennia marina* (Forssk.) Vierh. New Phytol 107: 317–326. 10.1111/j.1469-8137.1987.tb00183.x 33873850

[27] Mimura T, Kura-Hotta M, Tsujimura T, Ohnishi M, Miura M, et al (2003) Rapid increase of vacuolar volume in response to salt stress. Planta 216: 397–402. 12520330 10.1007/s00425-002-0878-2

[28] Feller IC, Whigham DF, O'Neill JP, McKee KM (1999) Effects of nutrient enrichment on within-stand nutrient cycling in mangrove ecosystems in Belize. Ecology 80: 2193–2205.

[29] Feller IC, McKee KL, Whigham DF, O'Neill JP (2003) Nitrogen vs. phosphorus limitation across an ecotonal gradient in a mangrove forest. Biogeochemistry 62: 145–175.

[30] Reef R, Feller IC, Lovelock CE (2010) Nutrition of mangroves. Tree Physiol 30: 1148–1160. 20566581 10.1093/treephys/tpq048

[31] Tam NFY, Wong YS (1998) Variations of soil nutrient and organic matter content in a subtropical mangrove ecosystem. Water Air Soil Poll 103: 245–261.

[32] Feller LC, Mckee KL, Whigham DF, O'Neill JP (2002) Nitrogen vs. phosphorus limitation across an ecotonal gradient in a mangrove forest. Biogeochemistry 00: 1–31.

[33] Whigham DF, Verhoeven JTA, Samarkin V, Megonigal PJ (2009) Responses of *Avicennia germinans* (Black Mangrove) and the Soil Microbial Community to Nitrogen Addition in a Hypersaline Wetland. Estuar Coast 32: 926–936.

[34] Yates EJ, Ashwath N, Midmore DJ (2002) Responses to nitrogen, phosphorus, potassium and sodium chloride by three mangrove species in pot culture. Trees 16: 120–125.

[35] Popp M, Lied W, Meyer AJ, Richter A, Schiller P, et al (1996) Sample preservation for determination of organic compounds: microwave versus freeze-drying. J Exp Bot 47: 1469–1473.

[36] Lovelock CE, Feller IC (2003) Photosynthetic performance and resource utilization of two mangrove species coexisting in a hypersaline scrub forest. Oecologia 134: 455–462. 12647116 10.1007/s00442-002-1118-y

[37] Nadini A, Salleo S (2000) Limitation of stomatal conductance by hydraulic traits: sensing or preventing xylem cavitation. Trees 15: 14–24.

[38] Lovelock CE, Ball MC, Choat B, Engelbrecht BMJ, Holbrook NM, et al (2006) Linking physiological processes with mangrove forest structure: phosphorus deficiency limits canopy development, hydraulic conductivity and photosynthetic carbon gain in dwarf *Rhizophora mangle* . Plant Cell Environ 29: 793–802. 17087463 10.1111/j.1365-3040.2005.01446.x

[39] Lin CC, Zhu TC, Liu L, Wang DL (2010) Influences of major nutrient elements on Pb accumulation of two crops from a Pb-contaminated soil. J Hazard Mater 174: 202–208. 19854574 10.1016/j.jhazmat.2009.09.137

[40] Leblebici Z, Aksoy A (2011) Growth and lead accumulation capacity of *Lemna minor* and *Spirodela polyrhiza* (Lemnaceae): interactions with nutrient enrichment. Water Air Soil Poll 214: 175–184. 10.1007/s11270-010-0413-1 PMC3003148 21258435

[41] Flowers TJ, Troke PF, Yeo AR (1977) The mechanism of salt tolerance in halophytes. Annu Rev Plant Physiol 28: 89–121.

[42] Shi GR, Cai QS, Liu CF, Wu L (2010) Silicon alleviates cadmium toxicity in peanut plants in relation to cadmium distribution and stimulation of antioxidative enzymes. J Plant Growth Regul 61: 45–52.

[43] Chen YP, Ye Y (2013) Growth and physiological responses of saplings of two mangrove species to intertidal elevation. Mar Ecol Prog Ser 482: 107–118.

[44] Ellison AM, Farnsworth EJ (1997) Simulated sea level change alters anatomy, physiology, growth, and reproduction of red mangrove (Rhizophora mangle L). Oecologia 112: 435–446. 28307619 10.1007/s004420050330

[45] Krauss KW, Allen JA (2003) Factors influencing the regeneration of the mangrove Bruguiera gymnorrhiza (L.) Lamk. on a tropical Pacific island. For Ecol Manag 176: 49–60.

[46] He FL (2004) Study on ecological restoration of mangrove ecosystem. Environmental Science and Technology 27: 81–83.

[47] Zhang ZH, Zhou RC, Tang T, Huang YL, Zhong Y, et al (2008) Genetic variation in central and peripheral populations of *Excoecaria agallocha* from Indo-West Pacific. Aquat Bot 89: 57–62.

[48] Li MY, Xiao Q, Pan JY, Wu J (2009) Natural products from semi-mangrove flora: source, chemistry and bioactivities. Nat Prod Rep 26(2): 281–298. 19177225 10.1039/b816245j

[49] Chandrasekaran M, Kannathasan K, Venkatesalu V, Prabhakaret K (2009) Antibacterial activity of some salt marsh halophytes and mangrove plants against methicillin resistant *Staphylococcus aureus* . World J Microb Biot 25(1): 155–160.

[50] Satyan RS, Aveek N, Eganathan P, Parida A (2010) Comparative histochemical localization of secondary metabolites in seed-raised and in vitro propagated plants of *Excoecaria agallocha* Linn. (Euphorbiaceae), the milky mangrove tree of historical significance. Biotech Histochem 85(5): 285–293. 19701827 10.1080/10520290903202478

[51] Zhang YB, Lin P, Wei XY, Zhuang TC (2008) Effects of salinity on microbial densities of soil in the dilution plate technique applied in mangrove areas. Acta Ecologoca Sinica 28: 1287–1295.

[52] Hoagland DR, Arnon DI (1950) The water-culture method for growing plants without soil. California Agricultural Experiment Station. Circular 347: 1–32.

[53] Hunt R (1978) Plant Growth Analysis. The Institute of Biology's Studies in Biology no. 96. Edward Arnold, London.

[54] Aebi H (1984) Catalase in vitro. Method Enzymol 105: 121–126. 10.1016/s0076-6879(84)05016-3 6727660

[55] Ye Y, Wong YS, Tam NFY (2005) Acclimation of a dominant mangrove plant (*Kandelia candel*) to soil texture and its response to canopy shade. Hydrobiologia 539: 109–119.

[56] Bates LS, Waldren RP, Teare ID (1973) Rapid Determination of Free Proline for Water Stress Studies. Plant Soil 39: 205–207.

[57] Abari AK, Nasr MH, Hojjati M, Bayat D (2011) Salt effects on seed germination and seedling emergency of two Acacia species. Afr J Plant Sci 5(1): 52–56.

[58] Khan MA, Gulzar S (2003) Light, salinity, and temperature effects on the seed germination of perennial grasses. Am J Bot 90(1): 131–134. 21659088 10.3732/ajb.90.1.131

[59] Benabderrahim MA, Haddad M, Hamza H, Ferchichi A (2011) Germination and emergence variability of alfalfa (*Medicago sativa* L.) landraces collected in Southern Tunisia oases. Span J Agric Res 9(1): 135–143.

[60] Werner JE, Finkelstein RR (1995) Arabidopsis mutants with reduced response to NaCl and osmostic stress. Physiol Plantarum 93: 659–666.

[61] Rehman S, Harris PJC, Bourne WF, Wilkin J (1997) The effect of sodium chloride on germination and the potassium and calcium contents of Acacia seeds. Seed Sci Technol 25(1): 45–57.

[62] Kaveh H, Nemati H, Farsi M, Jartoodeh SV (2011) How salinity affect germination and emergence of tomato lines. J Biol Environ Sci 5(15): 159–163.

[63] Parida AK, Das AB, Mittra B (2004) Effects of salt on growth, ion accumulation, photosynthesis and leaf anatomy of the mangrove, *Bruguiera parviflora* . Trees 18: 167–174.

[64] Srivastava DS, Jefferies RL (1995) The effect of salinity on the leaf and shoot demography of two arctic forage species. J Ecol 83: 421–430.

[65] Warwick NWM, Bailey PCE (1998) The effect of time of exposure to NaCl on leaf demography and growth for two non-halophytic wetland macrophytes, Potamogeton tricarinatus F. Muell. and A. Benn. Ex A. Benn. and Triglochin procera R. Br. Aquat Bot 62: 19–31.

[66] Ball MC, Sobrado MA (1999) Ecophysiology of mangroves: challenges in linking physiological processes with patterns in forest structure. In: Press MC, Scholes JD, Barker MG, (eds) Physiological plant ecology. The British Ecological Society, Blackwell Science, London 331–346.

[67] Cavalieri AJ, Huang AHC (1981) Accumulation of proline and glycinebetaine in *Spartina alterniflora* Loisel. in response to NaCl and nitrogen in the marsh. Oecologia 49: 224–228. 28309313 10.1007/BF00349192

[68] Naidoo G, McKee KL, Mendelssoh IA (1992) Anatomical and metabolic responses to waterlogging and salinity in *Spartina alterniflora* and *S. patens* (Poaceae). Am J Bot 79: 765–770.

[69] Feigin A (1985) Fertilization management of crops irrigated with saline water. Plant Soil 89: 285–299.

[70] Chow WS, Ball MC, Anderson JM (1990) Growth and photosynthetic responses of spinach to salinity: implications of K^+^ nutrition for salt tolerance. Aust J Plant Physiol 17: 563–567.

[71] Popp M, Larher F, Weigel P (1985) Osmotic adaptation in Australian mangroves. Vegetatio 61: 247–253.

[72] Parida AK, Das AB (2004) Effects of NaCl stress on nitrogen and phosphorous metabolism in a true mangrove *Bruguiera parviflora* grown under hydroponic culture. J Plant Physiol 161: 921–928. 15384403 10.1016/j.jplph.2003.11.006

[73] Rawson HM, Munns R (1984) Leaf expansion in sunflower as influenced by salinity and short-term changes in carbon fixation. Plant Cell Environ 7: 207–213.

[74] Clough BF, Sim RG (1989) Changes in gas exchange characteristics and water use efficiency of mangroves in response to salinity and vapour pressure deficit. Oecologia 79: 38–44. 28312810 10.1007/BF00378237

[75] Nandy P, Das S, Ghose M (2005) Relation of leaf micromorphology with photosynthesis and water efflux in some Indian mangroves. Acta Bot Croat 64(2): 331–340.

[76] Popp M (1984) Chemical composition of Australian mangroves. I. Inorganic ions and organic acids. Zeitschr Pflanzenphysiol 113: 395–409.

[77] Meloni DA, Oliva MA, Martinez CA, Cambraia J (2003) Photosynthesis and activity of superoxide dismutase, peroxidase and gluthathione reductase in cotton under salt stress. Environ Exp Bot 49: 69–76.

[78] Prochazkova D, Wilhelmova N (2007) Leaf senescence and activities of the antioxidant enzymes. Biol Plantarum 51: 401–406.

[79] Macfarlane GR, Burcrad MD (2001) Photosynthetic pigments and peroxidase activity as indicators of heavy metal stress in the grey mangrove, *Avicennia marina* (Forsk.) Vierh. Mar Pollut Bull 42(3): 233–240. 11381878 10.1016/s0025-326x(00)00147-8

[80] Dasgupta N, Nandy P, Das S (2011) Photosynthesis and antioxidative enzyme activities in five Indian mangroves with respect to their adaptability. Acta Physiol Plant 33: 803–810.

